# Multi-platform NMR Study of Pluripotent Stem Cells Unveils Complementary Metabolic Signatures towards Differentiation

**DOI:** 10.1038/s41598-020-58377-w

**Published:** 2020-01-31

**Authors:** Bénédicte Elena-Herrmann, Emilie Montellier, Anne Fages, Reut Bruck-Haimson, Arieh Moussaieff

**Affiliations:** 1 Univ Grenoble Alpes, CNRS, INSERM, IAB, Allée des Alpes, 38000 Grenoble, France; 2 Univ Lyon, CNRS, Université Claude Bernard Lyon 1, ENS de Lyon, Institut des Sciences Analytiques, UMR 5280, 5 rue de la Doua, 69100 Villeurbanne, France; 3 Institute for Drug Research, the Hebrew University, Jerusalem, Israel

**Keywords:** Biochemistry, Biological techniques, Biotechnology, Stem cells

## Abstract

Stem cells, poised to revolutionize current medicine, stand as major workhorses for monitoring changes in cell fate. Characterizing metabolic phenotypes is key to monitor in differentiating cells transcriptional and epigenetic shifts at a functional level and provides a non-genetic means to control cell specification. Expanding the arsenal of analytical tools for metabolic profiling of cell differentiation is therefore of importance. Here, we describe the metabolome of whole pluripotent stem cells (PSCs) using high‐resolution magic angle spinning (HR-MAS), a non-destructive approach for Nuclear Magnetic Resonance (NMR) analysis. The integrated ^1^H NMR analysis results in detection of metabolites of various groups, including energy metabolites, amino acids, choline derivatives and short chain fatty acids. It unveils new metabolites that discriminate PSCs from differentiated counterparts and directly measures substrates and co-factors of histone modifying enzymes, suggesting that NMR stands as a strategic technique for deciphering metabolic regulations of histone post-translational modifications. HR-MAS NMR analysis of whole PSCs complements the much used solution NMR of cell extracts. Altogether, our multi-platform NMR investigation provides a consolidated picture of PSC metabolic signatures and of metabolic pathways involved in differentiation.

## Introduction

Pluripotent stem cells (PSCs), i.e. cells that are able to produce all three basic body layers, provide unprecedented potential for disease research, drug screening, toxicology, and cell therapy development^1–4^. They are considered invaluable for the future of regenerative medicine^2,5–8^, and offer new horizons for personalized medicine, as they may be used to derive patient-specific functional somatic cells^9^. Importantly, metabolic perturbations are involved in the regulation of both the state of pluripotency^10–19^ and cell proliferation and viability^20–23^. In fact, the metabolic state of the cell is a hallmark of pluripotency, and a part of its pluripotency definition^24^. Thus, understanding metabolic perturbations during PSC differentiation may shed new light on mechanisms that drive and direct differentiation. Such understandings are of special interest given that for the commercialization of their differentiated derivatives, a non-genetic regulation of PSC differentiation will be of great value. Moreover, the metabolic microenvironment is central in the efforts to define conditions to culture large-scale quantities of PSCs. As one example, Tohyama *et al*. cultivated PSC cardiomyocyte derivatives in glucose-depleted culture medium containing abundant lactate and obtained cardiomyocytes of up to 99% purity that did not form tumors after transplantation^22^. Studies of PSC metabolism may thus contribute to our understanding of stem cell biology and developmental processes, as well as have important implications in future regenerative medicine.

A great portion of the literature that has evolved significantly in the last decade on PSC metabolism is focused on primary metabolism, and especially energy metabolism^12–14,16,17^. These studies suggest that changes in the primary metabolic network lead to remodeling of the epigenetic landscape that triggers the exit of pluripotency. Importantly, the metabolites that compose the primary metabolic network and are involved in the metabolic switch between pluripotency and differentiation are very susceptible to detection and quantification by NMR-based analysis, suggesting that NMR is an important tool for monitoring histone modifying metabolic processes in general, and PSC metabolic state in particular.

NMR offers critical insights into cell metabolism, and has previously been used for profiling many cell systems, including adult stem cells, with important clinical implications^25–29^. Several studies have notably described the metabolism of PSCs using solution NMR^12,28,30,31^. A comprehensive evaluation of the metabolic fingerprints provided by complementary NMR approaches relevant to the challenges of deciphering metabolic regulations of gene expression is yet to be described.

Traditionally, NMR metabolomics has relied on the analysis of solution samples obtained by extraction of biological materials. However, the analysis of cell extracts suffers from several disadvantages: i) the resulting metabolic profiles are partial and biased towards the extraction system used; ii) sample preparation may cause the loss or chemical modification of cellular components, limiting the information about *in vivo* metabolic events; iii) it may also introduce technical variability to the studied replicates. High-resolution magic angle spinning (HR-MAS) NMR spectroscopy enables the direct characterization of whole cells or tissues, allowing the simultaneous detection of polar and nonpolar metabolites, in a more global insight into their metabolic profiles. Rapid spinning of a sample at an angle of 54.7° (“magic angle”) relative to the applied magnetic field reduces line-broadening effects, hence resulting in well-resolved NMR spectra. The quality of the spectra obtained from HR-MAS experiments of intact biological tissues is comparable to that from aqueous extracts^32,33^. To date, no conclusive data has been shown to support a superior outcome of either HR-MAS or liquid phase NMR in non-targeted metabolic analysis of cells.

We have recently demonstrated the utilization of NMR-based global metabolic profiling of PSCs by characterization of the early metabolic shifts upon the exit of PSCs from the state of pluripotency, and the role of these shifts in the balance between pluripotency and differentiation^12^.

Here, we use PSC as a model for cell fate changes and concomitantly evaluate two NMR strategies for global fingerprinting of PSC metabolome: liquid phase analysis of aqueous extracts and HR-MAS NMR spectroscopy of whole cells. Metabolic profiles of PSCs are drawn and compared to those of cells that were differentiated toward a neuronal fate using both NMR platforms. Metabolic signatures of differentiation are unique to each NMR platform, underlining the complementarity of the two approaches. Importantly, HR-MAS NMR analysis unveils metabolites relevant to epigenetic control of gene expression.

## Materials and Methods

### ES cell culture and differentiation

CGR8 mouse ESCs (the kind gift of Dr. D. Aberdam) were maintained and neural differentiation carried out following Gambaro *et al*.^34^. Briefly, Cells were transferred to dishes with PFA-fixed NIH-3T3 feeder cells in gelatin coated (0.1% in phosphate buffered saline (PBS)). Cells were cultivated in GMEM medium containing 10% FCS (HyClone), 1 mM sodium pyruvate (Gibco BRL), 1% non-essential amino acids (Gibco BRL) and 0.1 μM 2-β mercaptoethanol (Sigma Aldrich). Non-differentiated ESCs were cultured and maintained in undifferentiated state with 10^3^ units/ml of Leukemia Inhibitory Factor (LIF). For differentiation, cells were seeded at low density (200,000 cells in 10 cm dish) with the following medium to allow differentiation: GMEM with 10% KSR (KnockOut serum replacement, Gibco BRL), 0.1 μM 2-β mercaptoethanol, 1 mM sodium pyruvate, 1% non-essential amino acids (NEAA) on fixed NIH-3T3 cells. Medium was replaced after 3 days in culture and every 2 days thereafter. At day 7, differentiated cells displayed long extensively branched neurites typical of neuronal morphology, at which point cells were harvested and analyzed. 37–42% of the cells were positive to CD57 in a FACS analysis (Supplementary Material Fig. S1A,B), and 80% presented typical neuronal dendrites and expressed neural markers on microscopic analysis (Fig. S1C–G).

### Immunohistochemistry

Cells were seeded on microscope slides in poly-l-ornithine coated plates (Sigma), washed twice with PBS and fixed with 4% paraformaldehyde for 15 min at room temperature. Following incubation with 1 mM glycine in PBS for 10 minutes, cells were permeabilized with 0.1% Triton X-100 for 5 min and blocked with 0.5% BSA, 2.5% donkey serum (Jackson) for 15 min. Cells were incubated with primary antibodies for 1 h, then washed and incubated with secondary antibodies for 1 h at room temperature. Samples were imaged on a microscope (Nikon eclipse Ti). Primary antibodies used for immunohistochemistry included donkey anti-mouse anti-Neun or anti-Tubulin β-III (1:100; both from Millipore). Secondary anti-donkey antibody (1:500; Invitrogen). Cells were counterstained for Hoechst 33342 (Invitrogen).

### Flow cytometry

Flow cytometry was carried out on a FACScan system using CellQuest software (BD Biosciences). Cells were washed in PBS, trypsinized, and centrifuged. Cell pellets were resuspended in PBS containing 2.5% BSA, filtered through a cell strainer to remove aggregates, and incubated with primary antibodies. As a neuroectodermal marker, cells were incubated with anti-CD57 (HNK1)^35^ for 40 min at 4 °C. Following wash in PBS, cells were incubated in rabbit anti-mouse secondary antibody (Dako). Cells were then washed twice, suspended in PBS with 1% FBS, and analyzed. Live, single cells were gated by GFP, PE, or side-scatter emission. Unlabeled cells and isotype controls were used to set gates for negative populations.

### NMR sample preparation

Cells were harvested by trypsinization and went through 3 passages on gelatin to exclude feeder cells. Cell pellets were washed in freshly prepared 0.9% NaCl solution in D_2_O. 1/20 of the cells were taken for cell counting and staining. Each replicate (~10 million cells) was then split to two samples of ~5 million cells: one for liquid extraction and one for HR-MAS.

Cells for HR-MAS NMR analysis were centrifuged at 300 *g* for 5 min at 4 °C, and washed in freshly prepared 0.9% NaCl solution in D_2_O. Cells were then gently homogenized in 30 μL 0.9% NaCl solution in D_2_O per sample, and transferred to HR-MAS disposable Kel-f inserts. Sealed inserts were then snap-frozen in liquid nitrogen and kept at −80 °C until analysis.

Cells for solution NMR analysis were centrifuged at 300 *g* for 5 min at 4 °C and washed in freshly prepared 0.9% NaCl solution in D_2_O (same washing solution as for the HR-MAS preparation). Cells were centrifuged and pellets were quenched in ice cold 60% MeOH, transferred to glass tubes and left for 30 minutes on ice. Samples were extracted in 300 μL of methanol/chloroform (2:1, v/v). Following Vortex mix for 1 min, samples were incubated for 15 minutes on ice, and went through ultra-sonication. 300 μL of chloroform/water (1:1, v/v) mix were added to sample and vortex-mixed again. Phase separation was carried out by centrifugation (1500 g, 20 min at 4 °C). Upper layer (aqueous phase) was transferred to a clean Eppendorf tube, and lower layer (lipophilic) to a separate glass tube, without the protein ring. The protein ring was then re-extracted at the same manner, and extracts from the protein ring were pooled with the main sample extracts and vacuum-dried. Samples were then snap-frozen in liquid nitrogen and kept at −80 °C until analysis. Dried aqueous extracts were then resuspended into 600 μL of phosphate buffer (pH = 7.2) in D_2_O containing 0.1 mM TSP (3-(trimethylsilyl)propionate-2,2,3,3-d4), and 550 μL of this final aqueous solution were then transferred into conventional 5 mm NMR tubes.

### NMR spectroscopy

All NMR experiments were performed on a Bruker Avance III spectrometer operating at 800.15 MHz (^1^H resonance frequency), equipped with either a 5 mm TXI solution NMR probe or a 4 mm HCP high-resolution MAS probe, and associated automated sample changers with cooling capacity for high-throughput acquisition (Bruker SampleJet and SamplePro for solution and HR-MAS NMR, respectively). Detailed NMR experimental parameters are provided in the Supplementary Material.

### NMR data analysis and statistics

Metabolites identifications were carried out from one-dimensional ^1^H profiles and multivariate signatures of statistical analysis (loadings), using ChenomX NMR Suite 8 (ChenomX Inc., Edmonton, Canada) and associated database of pure compounds, as well as the HMDB database^36^. ^1^H NMR shifts for detected metabolites are reported in Table S1. Multivariate statistical analyses were carried out using SIMCA 14 (Umetrics, Umea, Sweden) with centered variables. Principal component analysis (PCA) was first used to assess datasets homogeneity (for solution and HR-MAS data independently), exclude biological or technical outliers (samples well out of the 95% confidence interval), and identify primary sources of variance within the datasets. Sample classification models were then obtained using orthogonal partial least square discriminant analyses (OPLS-DA), with 7-fold cross-validation to select the adequate number of orthogonal components. Models were further validated by resampling 1000 times the models under the null hypothesis. Additionally, univariate unpaired two-tailed *t*-tests were performed in Matlab, using routines developed in-house, on clusters of variables obtained following the statistical recoupling of variables (SRV) method^37^. The false discovery rate (FDR) was controlled at a 5% level using the Benjamini-Hochberg procedure.

## Results and Discussion

**Figure 1:**
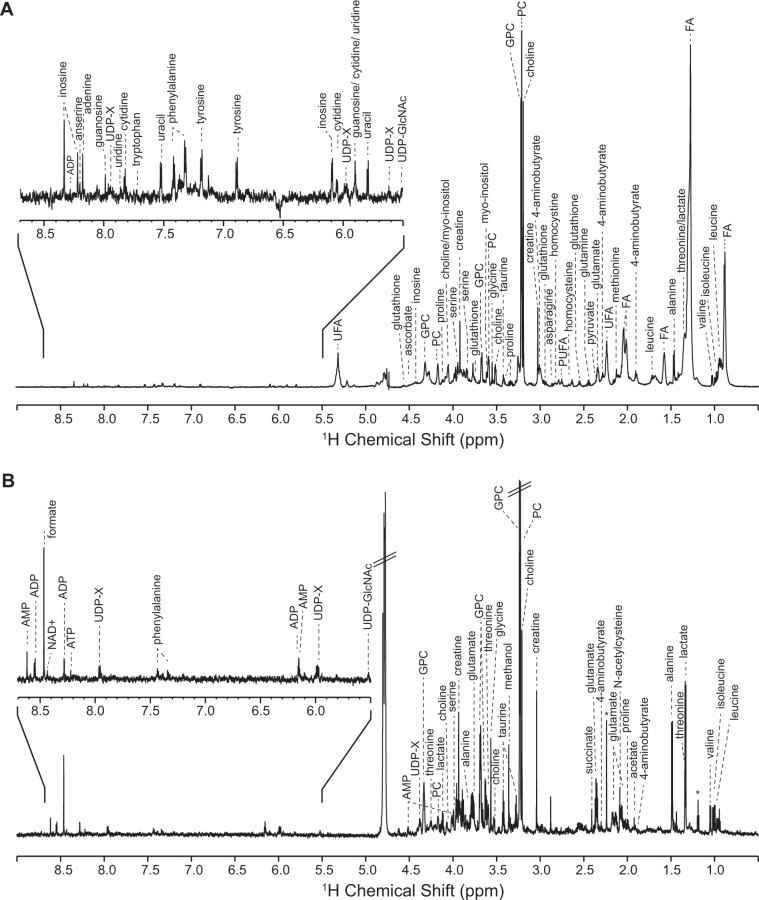
Representative ^1^H NMR metabolic fingerprints of CGR8 ESCs, as recorded (**A**) for whole cells by HR-MAS spectroscopy, and (**B**) for aqueous extracts by conventional solution NMR. GPC: glycerophosphocholine; PC: phosphocholine; FA: fatty acids; UFA: unsaturated fatty acids; PUFA: polyunsaturated FA; UDP-GlcNAc: UDP-N-acetylglucosamine; UDP-X: UDP-galactose/UDP-glucose/UDP-glucuronate/UDP-GlcNAc; *solvent trace: ethanol.

PSCs either maintained in an undifferentiated state or after undergoing neural differentiation (Supplementary Material Fig. S1) were characterized by NMR using two different analytical platforms, solution analysis of aqueous extracts and HR-MAS analysis of whole cells, in order to decipher metabolic phenotypes associated with PSCs and their differentiation. One-dimensional ^1^H NMR profiles were obtained from 13 cell samples, using both platforms (Fig. 1), which allowed to identify unambiguously a total of 56 metabolites in either type of cells, PSCs or differentiated cells (Table 1). Of these, 21 metabolites were identified by a single analytical approach: 15 metabolites were detected by HR-MAS NMR of whole cells only, while 6 were found by solution analysis of aqueous extracts exclusively.

**Table 1:** List of identified metabolites as detected respectively by HR-MAS NMR analysis of whole cells, or from aqueous extracts by conventional solution NMR acquisition, for PSC and differentiated (Diff) cells samples.

	Metabolite	NMR Platform	Cell Types
1	4-aminobutyrate	HR-MAS, Aq. extracts	Diff, PSC
2	Acetate	HR-MAS, Aq. extracts	Diff, PSC
3	Acetylcholine	HR-MAS, Aq. extracts	Diff, PSC
4	ADP	HR-MAS, Aq. extracts	Diff^a^, PSC
5	Alanine	HR-MAS, Aq. extracts	Diff, PSC
6	AMP	HR-MAS, Aq. extracts	Diff, PSC
7	Ascorbate	HR-MAS, Aq. extracts	Diff, PSC
8	Choline	HR-MAS, Aq. extracts	Diff, PSC
9	Creatine	HR-MAS, Aq. extracts	Diff, PSC
10	Formate	HR-MAS, Aq. extracts	Diff, PSC
11	Glutamate	HR-MAS, Aq. extracts	Diff, PSC
12	Glutathione	HR-MAS, Aq. extracts	Diff, PSC
13	Glycerophosphocholine (GPC)	HR-MAS, Aq. extracts	Diff, PSC
14	Glycine	HR-MAS, Aq. extracts	Diff, PSC
15	Inosine	HR-MAS, Aq. extracts	Diff, PSC^b^
16	Isocitrate	HR-MAS, Aq. extracts	Diff, PSC
17	Isoleucine	HR-MAS, Aq. extracts	Diff, PSC
18	L-Lactate	HR-MAS, Aq. extracts	Diff, PSC
19	Leucine	HR-MAS, Aq. extracts	Diff, PSC
20	Myo-inositol	HR-MAS, Aq. extracts	Diff, PSC
21	NAD+	HR-MAS, Aq. extracts	Diff, PSC^a^
22	Phenylalanine	HR-MAS, Aq. extracts	Diff, PSC
23	Phosphocholine (PC)	HR-MAS, Aq. extracts	Diff, PSC
24	Proline	HR-MAS, Aq. extracts	Diff, PSC
25	Pyruvate	HR-MAS, Aq. extracts	Diff, PSC
26	Serine	HR-MAS, Aq. extracts	Diff, PSC
27	Threonine	HR-MAS, Aq. extracts	Diff, PSC
28	Tyrosine	HR-MAS, Aq. extracts	Diff, PSC
29	UDP-galactose	HR-MAS, Aq. extracts	Diff, PSC
30	UDP-glucose	HR-MAS, Aq. extracts	Diff, PSC
31	UDP-glucuronate	HR-MAS, Aq. extracts	Diff, PSC
32	UDP-N-acetylglucosamine	HR-MAS, Aq. extracts	Diff, PSC
33	Valine	HR-MAS, Aq. extracts	Diff, PSC
34	Glycerol	HR-MAS, Aq. extracts	Diff
35	Taurine	HR-MAS, Aq. extracts	PSC
36	Adenine	HR-MAS	Diff, PSC
37	Asparagine	HR-MAS	Diff, PSC
38	Butyrate	HR-MAS	Diff, PSC
39	Cytidine	HR-MAS	Diff, PSC
40	Glutamine	HR-MAS	Diff, PSC
41	Guanosine	HR-MAS	Diff, PSC
42	Homocysteine	HR-MAS	Diff, PSC
43	Homocystine	HR-MAS	Diff, PSC
44	Methionine	HR-MAS	Diff, PSC
45	Nicotinamide	HR-MAS	Diff, PSC
46	Uracil	HR-MAS	Diff, PSC
47	Uridine	HR-MAS	Diff, PSC
48	Citrate	HR-MAS	Diff
49	N-acetylglutamine	HR-MAS	Diff
50	Tryptophan	HR-MAS	Diff
51	Arginine	Aq. Extracts	Diff, PSC
52	Aspartate	Aq. Extracts	Diff, PSC
53	GTP	Aq. Extracts	Diff, PSC
54	N-acetylcysteine	Aq. Extracts	Diff, PSC
55	Succinate	Aq. extracts	Diff, PSC
56	UMP	Aq. extracts	Diff

^a^Only in aqueous extracts; ^b^by HR-MAS only.

**Figure 2:**
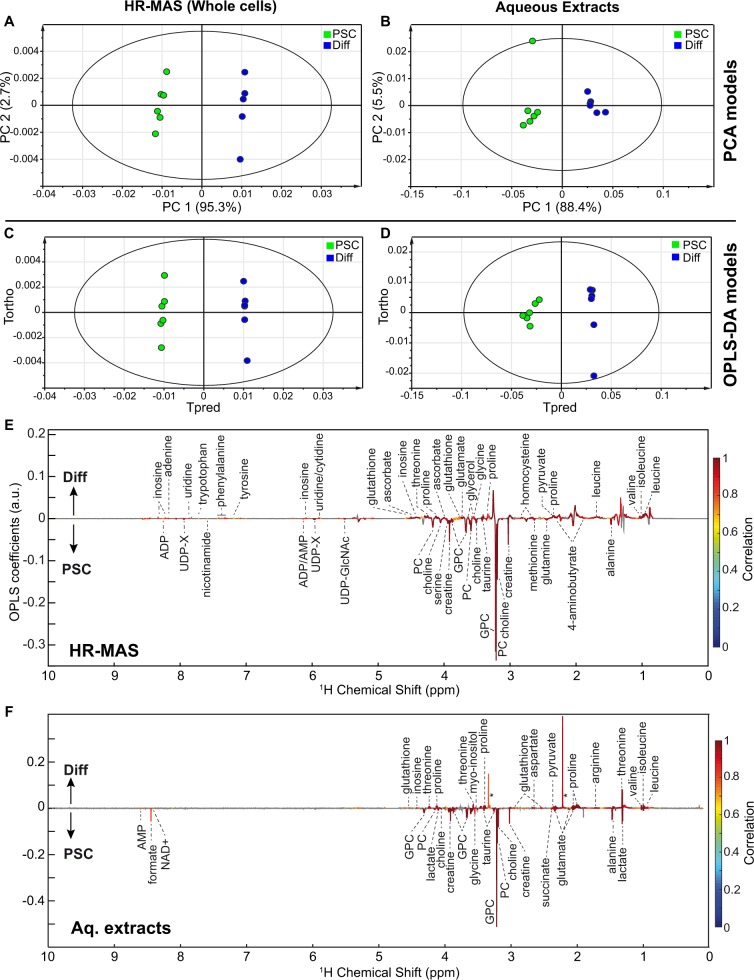
Multivariate statistical analyses of ^1^H NMR fingerprints discriminates PSC and differentiated (Diff) cells. HR-MAS investigation of whole cells (**A**,**C**,**E**) and solution NMR analysis of aqueous extracts (**B**,**D**,**F**): (**A**,**B**) Score plots from principal component analysis (PCA) (model A: N = 12, 4 components, R^2^X = 0.992, Q^2^ = 0.978; model B: N = 12, 2 components, R^2^X = 0.938, Q^2^ = 0.828). (**C**,**D**) Score plots and (**E**–**F**) associated loadings from OPLS discriminant analysis (model C: N = 12, 1 predictive +1 orthogonal components, R^2^X = 0.979, R^2^Y = 0.999, Q^2^ = 0.998; model D: N = 12, 1 predictive +1 orthogonal components, R^2^X = 0.923, R^2^Y = 0.984, Q^2^ = 0.963). GPC: glycerophosphocholine; PC: phosphocholine; UDP-GlcNAc: UDP-N-acetylglucosamine; UDP-X: UDP-galactose/UDP-glucose/UDP-glucuronate/UDP- GlcNAc; *solvent traces: methanol, acetone.

**Figure 3:**
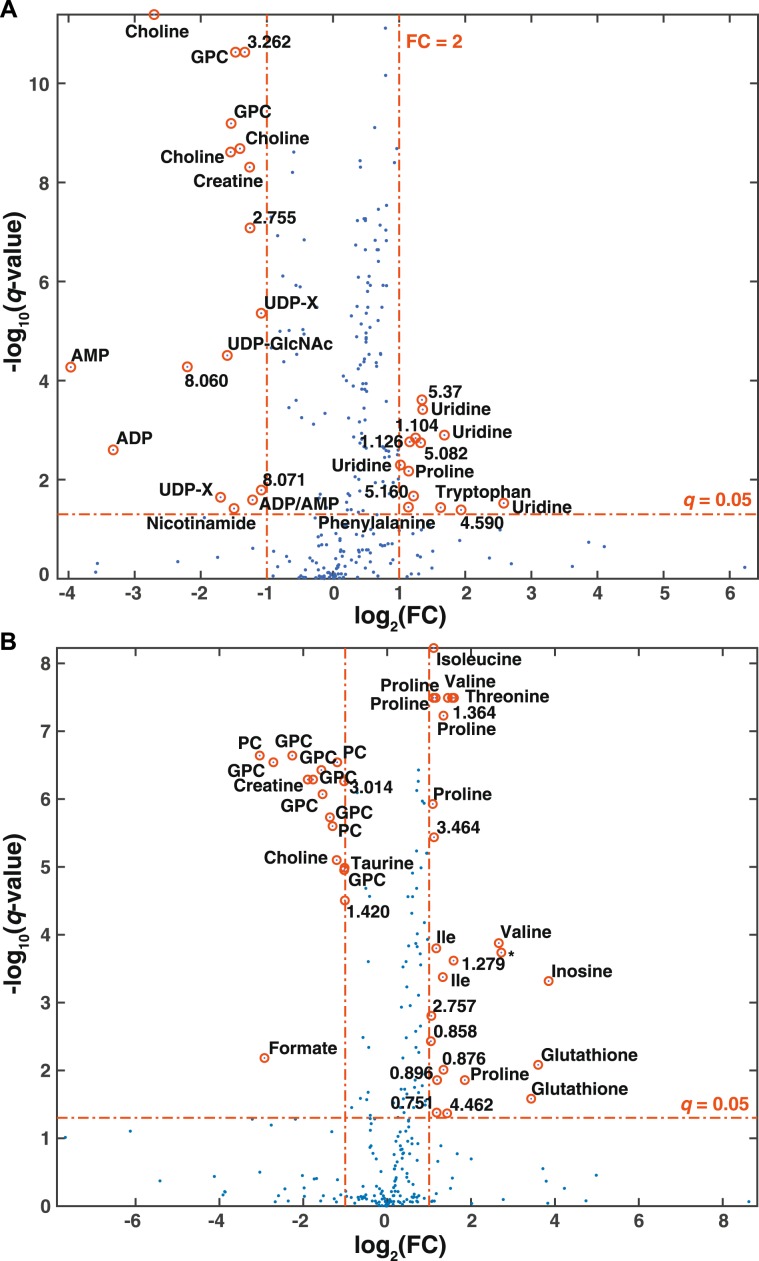
Volcano plot of metabolic alterations between differentiated and PSC cells as measured by (**A**) HR-MAS analysis of whole cells and (**B**) solution NMR of aqueous extracts. Red lines reflect the filtering criteria (|log_2_(FC)| = 1 and *q* = 0.05). *q*-values are corrected *p*-values with the Benjamini-Hochberg procedure to control the false discovery rate. GPC: glycerophosphocholine; PC: phosphocholine; UDP-GlcNAc: UDP-N-acetylglucosamine; UDP-X: UDP-galactose/UDP-glucose/UDP-glucuronate/UDP- GlcNAc; Ile: isoleucine; *acetone.

An initial principal component analysis (PCA) of the corresponding datasets (data not shown) led to the exclusion of two severe outliers, i.e. one solution and one HR-MAS sample, and subsequent multivariate analyses were carried out on 12 samples for each analytical cohort. For both investigations of extracts and whole cells, PCA score plots prominently revealed a discrimination of samples according to their differentiation state (Fig. 2A,B) along the first principal component (PC1). This discrimination was then further examined using orthogonal partial least squares (OPLS) supervised models (Fig. 2C–F), in which the loadings characterized metabolic signatures of neuroectodermal differentiation. A volcano analysis revealed metabolites with fold-changes larger than two, and significant variations (*q* < 0.05) as estimated from univariate testing with FDR correction (Fig. 3, Table S2). Of these, 12 metabolites were identified in the liquid analysis, and 13 in the analysis of whole cells (Table S2).

Previous work by us and others suggests dramatic changes in metabolite content of PSCs upon the exit of pluripotency. Taken together, these previous studies suggest metabolic regulation as an important segment of the molecular circuitry that determines cell fate^12,13,16,19,24,38,39^. Developing an arsenal of analytical approaches to monitor metabolic shifts in differentiating cells is thus of great value for understanding developmental and pathological processes, as well as stem cell biology for their large-scale cultivation and for generation of differentiation protocols. It is important to note that a major caveat when interpreting information obtained from metabolic profiling experiments results from processing of samples. Solvents and protocols used in the pre-analytical phase are critical determinants of the nature of compounds that compose the metabolic profiles. Besides causing an obvious bias towards the detection of a specific segment of the metabolome, sample processing is also suggested to exert dramatic changes and great variability in the content and composition of the metabolome of intact cells, as well as hamper identification coverage^40–43^. HR-MAS NMR offers a direct analysis of whole cells, with almost no sample processing, providing means for robust and high throughput monitoring of cells. Trypsinization may affect the cells metabolic profiles. Sample freezing and fast spinning are also known to partially disrupt cellular membranes^44^. Yet, this should not impact the comparative analysis of different cell cultures. Another important aspect when considering metabolism of stem cells is their rarity, low availability and high cost, in many cases. A non-destructive approach such as HR-MAS NMR for metabolic profiling of these cells is therefore of considerable interest.

**Figure 4:**
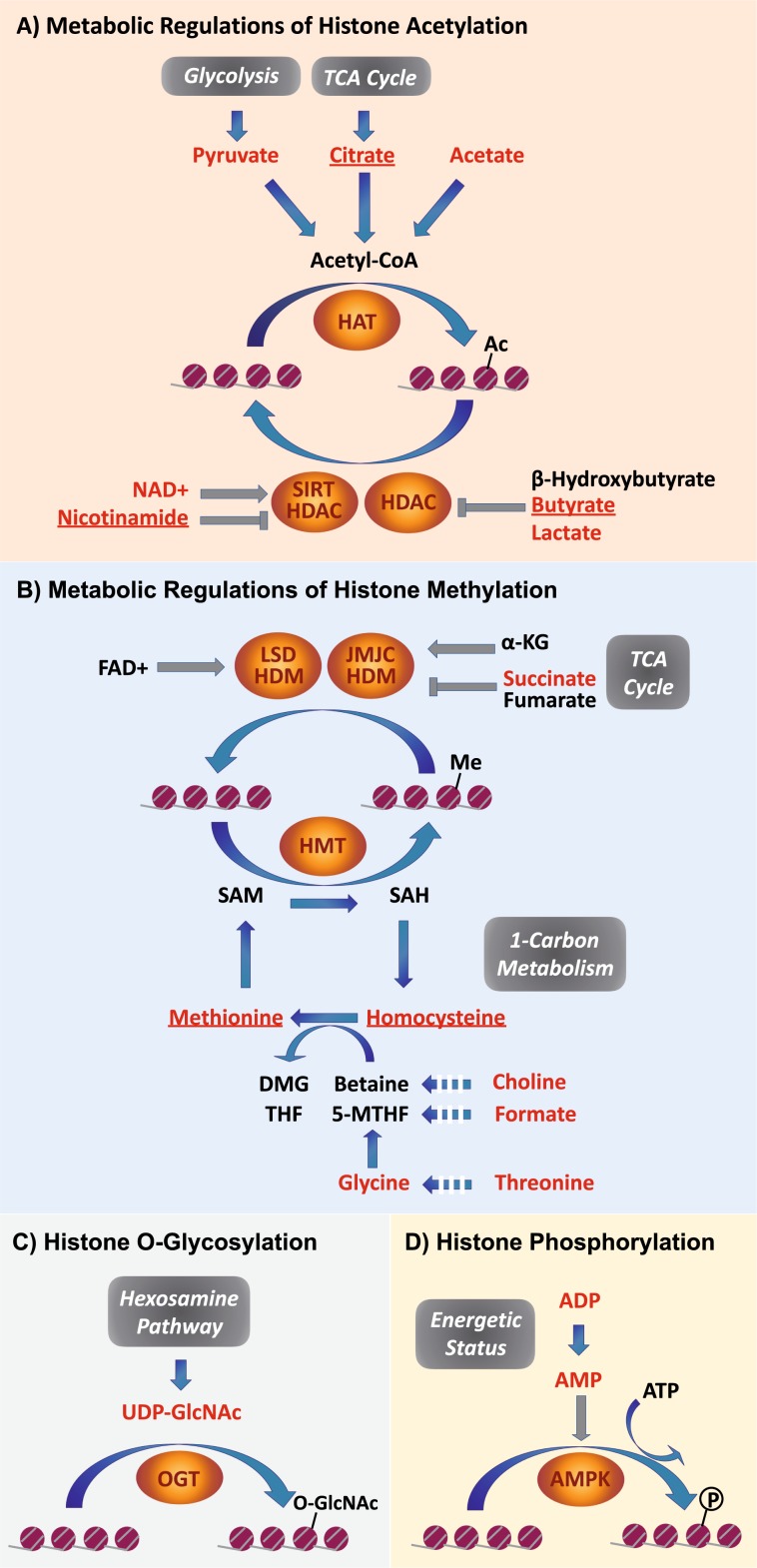
Schematic pathways for metabolic regulations of histones post-translational modifications: actetylation (**A**), methylation (**B**), O-glycosylation (**C**) and phosphorylation (**D**). Metabolites detected in PSC or differentiated cells by NMR are marked in red. Those detected by HR-MAS only in our experiments are additionally underlined. Abbrevations: 5-MTHF: 5-methyltetrahydrofolate; αKG: α-ketoglutarate; DMG: dimethylglycine; SAH: S-adenosyl-l-homocysteine; SAM: S-adenosyl-l-methionine; THF: tetrahydrofolate; UDP-GlcNAc: UDP-N-acetylglucosamine; AMPK: AMP-activated protein kinase; HAT: histone acetyltransferases; HDAC: histone deacetylases; HMT: histone methyltransferases; JMJC HDM: JmjC family of histone demethylases; LSD HMD: LSD family of histone demethylases; OGT: O-Linked N-Acetylglucosamine Transferase; SIRT HDAC: sirtuin family of histone deacetylases.

In the current study we present for the first time metabolic profiles of whole PSCs and differentiating cells, as obtained by HR-MAS NMR investigation. HR-MAS profiles are not biased towards a specific extraction system, and our HR-MAS analysis revealed metabolites that could not be detected and identified by a parallel analysis of the same cells by commonly used NMR solution analysis. These constitute almost 30% of the identified metabolites and include: adenine, asparagine, butyrate, citrate, cytidine, glutamine, guanosine, homocysteine, homocystine, methionine, N-acetylglutamine, nicotinamide, tryptophan, uracil, and uridine (Table 1). An important portion of the identified metabolites are involved in epigenetic regulation of the genes transcription programs, i.e. an ensemble of metabolic pathways that include substrates and co-factors of chromatin modifying enzymes (Fig. 4). Our data reveal that solution and HR-MAS NMR approaches provide means for the generation of a versatile arsenal of metabolites that contribute to shifts in the epigenetic landscape, and mainly post-translational modifications (PTMs) of histones:

(i) Acetylation: Acetyl-CoA (Ac-CoA), the substrate of histone acetylation by acetyltransferases (HAT), represents a major metabolic junction. It may be synthetized from 3 precursors: pyruvate, citrate and acetate. While Ac-CoA is not readily detected by our NMR methods, the detection and quantification of acetate, citrate and pyruvate is straightforward. Moreover, NMR provides means for monitoring the metabolic regulators of histone deacetylases: NAD + (sirtuin histone deacetylase cofactor), nicotinamide (sirtuin histone deacetylase inhibitor), and two major metabolic HDAC inhibitors: butyrate and lactate. Of note, citrate, nicotinamide and butyrate are exclusively detected by the HR-MAS platform, underscoring its unique value as means to monitor metabolic perturbations that underlie changes in the epigenetic landscape.
(ii) Methylation: The cellular methyl donor in mammals is S-adenosylmethionine (SAM), synthesized from methionine and ATP. SAM is a metabolic junction of the one carbon metabolism pathway, crossing the folate and methionine pathways^13,45^. This metabolic crossroad is extremely important in retaining the pluripotency/differentiation balance, as extensive DNA methylation characterizes the exit of naive pluripotency state in ESC^46,47^. We detected key metabolites linked to the one carbon pathway using a combination of the two NMR platforms. Despite the fact that SAM, the substrate of histone methyltransferases (HMT), is not detected in our experiments, other metabolites linked to the one-carbon metabolism are monitored, including methionine (SAM precursor) and homocysteine (intermediate of SAM recycling once methylation occurs). Importantly, these metabolites are also only detected by HR-MAS. Other metabolites involved in conversion of methionine to SAM are also found in our analysis: choline, the precursor of betaine, as well as formate, threonine and glycine that are precursors of 5-methyltetrahydrofolate (5-MTHF). These provide means for the recycling of SAM. Succinate, an inhibitor of JmjC family of histone demethylases (JMJC HDM) is detected by solution NMR. These metabolites are also involved in the direct DNA methylation catalyzed by DNA methyltransferases (DNMT).
(iii) O-glycosylation (O-GlcNAcylation)^48^: interestingly, UDP-GlcNAc, which is the substrate of O-Linked N-Acetylglucosamine Transferase (OGT) to catalyze histone O-glycosylation is detected by both NMR approaches. Glutamine, an amino acid necessary for UDP-GlcNAc synthesis is observed by HR-MAS only.
(iv) Phosphorylation: AMP and ADP, which define the energetic status of the cell, are readily detected in our experiments. The kinase AMPK, a metabolic sensor of low cellular energy, is activated by AMP and is known to phosphorylate histones.

Overall, these observations highlight not only the broad scope of metabolites involved in chromatin modifying pathways by NMR approaches, but the particular importance of HR-MAS investigation of whole cells to probe metabolites regulating histone modifications.

A second aspect of our study is the evaluation of the metabolome of PSCs following neural differentiation (Fig. 2) using both HR-MAS and NMR of aqueous extracts. Metabolic signatures defined by the two approaches partially overlap, notably highlighting choline, phosphocholine (PC) and glycerophosphocholine (GPC) levels, involved in the synthesis of phospholipids, are significantly impacted by PSC differentiation, with their levels dropping by more than a factor 2 (Fig. 3, Table S2). These results are in perfect alignment with our previous results that point to phosphocholine as a major metabolic signature of differentiating PSCs^12^. Enhanced choline metabolism is a hallmark of cancer cells metabolism^49^, and has been demonstrated as a marker of pluripotency^28,30^. The choline metabolic signature shared by PSCs and cancer cells is not surprising, and reflects a general notion that these cells are metabolically close, with the Warburg effect being the first and most prominent example of such similarities. High creatine levels, observed in PSCs as compared with differentiated cells by both approaches may also reflect the high energetic demand (and ATP production) associated with proliferation, as creatine is the product of the phosphocreatine/creatine kinase system for ATP regeneration, which fuels the cells.

Notably, a considerable number of metabolic features of neural differentiation were uniquely detected by HR-MAS NMR: the accumulation of uridine, phenylalanine, tryptophan with fold changes (FC) larger than 2 (Fig. 3, Table S2), as well as significantly increased levels of adenine, tyrosine, ascorbate, homocysteine (Fig. 2). In contrast, we observe strong diminution of UDP-N-acetylglucosamine, ADP, AMP and nicotinamide (FC < 0.5) (Table S2), while serine, methionine, glutamine and 4-aminobutyrate show significant decreases, but with lower magnitude. Conversely, solution NMR reveals a few metabolites with significant variations along differentiation that were not perceived by the HR-MAS analysis. These included decreased concentrations of aspartate, arginine and myo-inositol increases, as well as taurine, formate, succinate and lactate associated with PSCs differentiation (Fig. 2).

The two NMR approaches provide consistent and complementary snapshots of amino-acid metabolism during PSC differentiation. Overall, proline, threonine and branched-chain amino-acids (isoleucine, leucine and valine) significantly discriminate differentiated from non-differentiated PSCs when tested by both approaches (Fig. 2), with large positive fold changes detected for proline and threonine (Fig. 3, Table S2). Proline was already shown to trigger PSCs differentiation^50^. HR-MAS NMR also presents tryptophan and phenylalanine as major discriminating metabolites between differentiating and non-differentiating cells.

PSCs and differentiated cells show altered levels of metabolites regulating histone PTM. Indeed, nicotinamide, formate (both with large fold change), methionine, succinate and lactate are enriched in PSCs whereas homocysteine, threonine are enriched in differentiated cells (Figs. 2 and 3). Modulation of these metabolic levels might affect epigenetic landscapes (Fig. 4) and play a role in the process of PSCs maintenance or neuronal differentiation. Accumulation of uridine diphosphate N-acetylglucosamine (UDP-GlcNAc) clearly discriminates differentiated from non-differentiated PSCs as seen in whole cells by HR-MAS NMR (Fig. 3). O-GlcNAc contributes to the landscape of histones modifications and regulates transcription factors involved in pluripotency maintenance that delay mouse ESC differentiation^51,52^.

Taken together, our work suggests that HR-MAS NMR analysis is highly complementary to liquid phase NMR for the study of epigenetic regulation by metabolic shifts and of the metabolism of differentiating cells. Our data also suggest that HR-MAS NMR may serve as a valuable non-destructive approach for the study and monitoring of PSCs and potentially other stem cells. Our analyses of the metabolic signatures of differentiating PSCs align with earlier work that suggests metabolic remodeling of differentiating PSCs, and especially to neural fate. PSC serve here as a model for cell fate changes. Our work suggests that many important shifts relevant to epigenetic regulations of gene expression may be monitored by HR-MAS, thus opening opportunities for a broad range of mechanistic studies based on non-invasive metabolic analysis of SCs. Future use of SCs for regenerative medicine may profit greatly from the optimization of analytical approaches for the monitoring SC metabolism.

## Supplementary Information

Supplementary Information.
