# Supplementary material for: Multi-platform NMR Study of Pluripotent Stem Cells Unveils Complementary Metabolic Signatures towards Differentiation
Source: Sci Rep. 2020 Jan 31;10:1622. doi: 10.1038/s41598-020-58377-w (PMC6994671; doi:10.1038/s41598-020-58377-w)
Supplement: Supplementary file 1 — Supplementary Information. [file 41598_2020_58377_MOESM1_ESM.pdf]

Supplementary Material to:

Multi-platform NMR Study of Pluripotent Stem Cells Unveils Complementary Metabolic Signatures towards Differentiation.

Bénédicte Elena-Herrmann[1,2*], Emilie Montellier[1], Anne Fages[2], Reut Bruck-Haimson[3], and Arieh Moussaieff[3*]

[1] *Institute for Advanced Biosciences, CNRS UMR 5309, INSERM U1209, Université Grenoble-Alpes, Grenoble, France.*

[2] *Université de Lyon, Institut des Sciences Analytiques, UMR 5280, CNRS, Université Lyon 1, ENS Lyon, 5, rue de la Doua, F-69100 Villeurbanne, France.*

[3] *Institute for Drug Research, the Hebrew University, Jerusalem, Israel.*

Content:

-Detailed NMR experimental parameters

-**Figure S1:** Cell morphology and expression of neuronal markers at day 7 of PSCs differentiation

-**Table S1:** List of identified metabolites and corresponding ^{1}H NMR shifts

-**Table S2:** List of discriminant metabolites from volcano analysis (differentiated vs. PSC cells)

NMR Spectroscopy: Experimental Parameters

All samples were kept at 4°C during idle time before NMR acquisition. All NMR experiments were carried out at 27 °C. For solution samples, ^{1}H metabolic profiles were recorded using a conventional NOESY experiment with water presaturation (Bruker pulse program noesygppr1d) using a spectral width of 16026 Hz and acquisition time of 1.5 s, a mixing time of 10 ms, and a recycle delay of 2 s for each of the 512 scans that were co-added. The 90°s pulse length was automatically calibrated on each sample at around 10.7 μs after automatic shimming and tuning. For HR-MAS NMR acquisition, 30 μL disposable Kel-f inserts were used together with standard 4 mm rotors. A spinning frequency of 4200 Hz was used to ensure that potential distortions from water spinning sidebands fall outside the useful spectral range. Shims were adjusted on a full rotor (without inserts) of 1% chloroform in acetone-d6, and no further adjustment was needed on individual cell samples of restricted volume. HR-MAS ^{1}H metabolic profiles were obtained using a Carr-Purcell-Meiboom-Gill experiment with water presaturation (pulse program cpmgpr1d) with a spectral width of 16026 Hz, acquisition time of 1.4 s, and a recycle delay of 2 s for each of the 256 scans that were co-added. A train of 128 spin echoes was used for the CPMG filter, corresponding to a total length of 80 ms. Note that for solution samples, the use of CPMG was not required, as high molecular weight content was previously discarded through the cell extraction procedure. NMR free induction decays were multiplied by an exponential function corresponding to a line broadening of 0.3 Hz and 1 Hz prior to Fourier transform for solution and HR-MAS data, respectively. All spectra were manually phased and referenced to either the signal of TSP (cell extracts) at -0.016 ppm, or the alanine doublet (whole cells) at 1.47 ppm. The residual water signal was excluded (range 4.7-4.9 ppm for aqueous extracts, and 4.6-4.95 ppm for HR-MAS spectra),

and datasets were bucketed over the spectral range [0.1-10 ppm] or [0.5-10 ppm] for solution and HR-MAS spectra respectively into variables of 0.001 ppm width using the AMIX Software (Bruker GmbH). Neither extracts nor HR-MAS datasets required any additional alignment procedure.

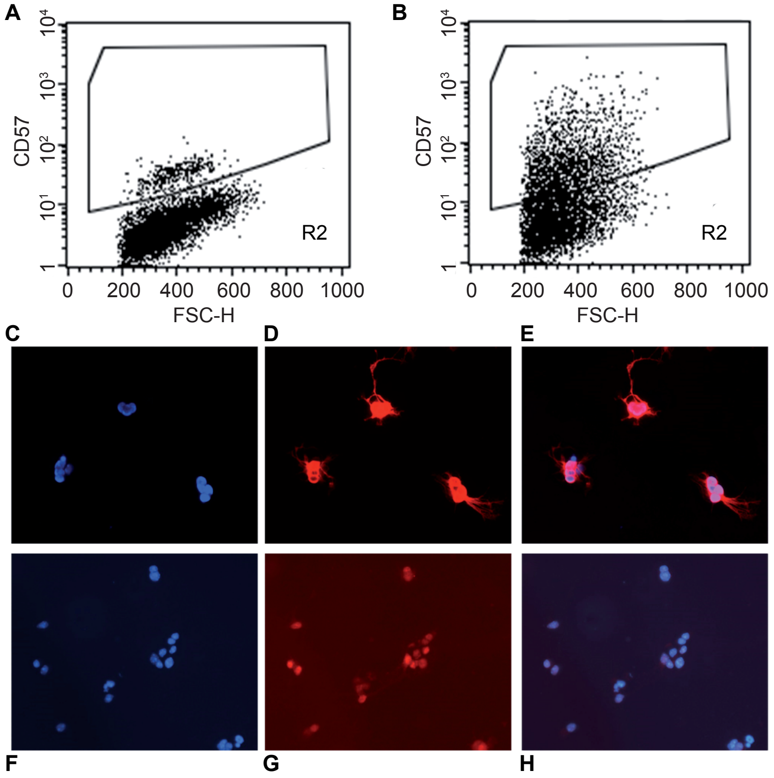

Figure S1: Cells at day 7 of differentiation exhibit neuronal typical morphology and express neuronal markers. FACS analysis following staining for CD57 for (A) pluripotent CGR8 ESCs and (B) CGR8 ESCs following one week of differentiation following Gambaro et al [34]. Differentiated cells were stained for: Hoechst nuclear staining (C,F); anti- Tubulin (D), or anti-Neun (G) and overlays of Hoechst with anti-Tubulin (E) or anti-Neun (H). Images were taken under x 40 magnification.

Table S1: List of identified metabolites and corresponding ¹H NMR shifts as detected respectively by HR-MAS NMR analysis of whole cells, or from aqueous extracts by conventional solution NMR acquisition, for PSC and/or differentiated cells samples.

	Metabolite	NMR Platform	^{1}H NMR Shift (ppm/multiplicity)
1	4-aminobutyrate	HR-MAS, Aq. extracts	3.01(t); 2.29(t); 1.89(m)
2	Acetate	HR-MAS, Aq. extracts	1.91(s)
3	Acetylcholine	HR-MAS, Aq. extracts	4.535(m); 3.215(s); 2.14(s)
4	ADP	HR-MAS, Aq. extracts	8.51(s); 8.255(s); 6.135(d)
5	Alanine	HR-MAS, Aq. extracts	3.77(a); 1.47(d)
6	AMP	HR-MAS, Aq. extracts	8.58(s); 8.245(s); 6.115(d)
7	Ascorbate	HR-MAS, Aq. extracts	4.50(d); 4.01(m)
8	Choline	HR-MAS, Aq. extracts	4.05(m); 3.51(m); 3.19(s)
9	Creatine	HR-MAS, Aq. extracts	3.92(s); 3.025(s)
10	Formate	HR-MAS, Aq. extracts	8.445(s)
11	Glutamate	HR-MAS, Aq. extracts	3.75(dd); 2.34(m)
12	Glutathione	HR-MAS, Aq. extracts	4.56(dd); 3.765(m); 2.945(m); 2.965(dd); 2.92(dd); 2.55(m); 2.16(m)
13	Glycerophosphocholine (GPC)	HR-MAS, Aq. extracts	3.67(m); 3.61(dd); 3.22(s)
14	Glycine	HR-MAS, Aq. extracts	3.55(s)
15	Inosine	HR-MAS, Aq. extracts	8.335(s); 8.22(s); 6.09(d)
16	Isocitrate	HR-MAS, Aq. extracts	2.985(m); 2.52(dd); 2.47(dd)
17	Isoleucine	HR-MAS, Aq. extracts	3.66(d); 1.00(d); 0.93(t)
18	L-Lactate	HR-MAS, Aq. extracts	4.115(q); 1.315(d)
19	Leucine	HR-MAS, Aq. extracts	0.955(d); 0.945(d)
20	Myo-inositol	HR-MAS, Aq. extracts	4.05(t); 3.61(t); 3.525(dd); 3.27(t)
21	NAD+	HR-MAS, Aq. extracts	9.33(s); 9.14(d); 8.84(m); 8.41(d); 8.17(s); 6.03(d)
22	Phenylalanine	HR-MAS, Aq. extracts	7.42(m); 7.37(m); 7.32(d); 3.12(dd)
23	Phosphocholine (PC)	HR-MAS, Aq. extracts	4.165(m); 3.59(m); 3.21(s)
24	Proline	HR-MAS, Aq. extracts	4.12(dd); 3.42(m); 3.33(m); 2.34(m); 2.06(m); 1.98(m)
25	Pyruvate	HR-MAS, Aq. extracts	2.36(s)
26	Serine	HR-MAS, Aq. extracts	3.98(dd); 3.94(dd); 3.84(dd)
27	Threonine	HR-MAS, Aq. extracts	4.25(m); 3.58(d); 1.32(d)
28	Tyrosine	HR-MAS, Aq. extracts	7.185(m); 6.89(d)
29	UDP-galactose	HR-MAS, Aq. extracts	7.94(d); 5.98(d); 5.96d); 5.62(dd)
30	UDP-glucose	HR-MAS, Aq. extracts	7.945(d); 5.975(d); 5.96(d); 5.59(dd)
31	UDP-glucuronate	HR-MAS, Aq. extracts	7.93(d); 5.985(d); 5.98(d); 5.615(dd)
32	UDP-N-acetylglucosamine	HR-MAS, Aq. extracts	8.33(d); 7.93(d); 5.97(d); 5.96(d); 5.505(dd)
33	Valine	HR-MAS, Aq. extracts	3.60(d); 1.03(d); 0.98(d)
34	Glycerol	HR-MAS, Aq. extracts	3.64(dd); 3.55(dd)
35	Taurine	HR-MAS, Aq. extracts	3.405(t); 3.26(t)

36	Adenine	HR-MAS	8.22(s); 8.18(s)
37	Asparagine	HR-MAS	4.00(dd); 2.945(dd); 2.85(dd)
38	Butyrate	HR-MAS	2.145(t); 1.54(m)
39	Cytidine	HR-MAS	7.825(d); 6.05(d); 5.895(f);
40	Glutamine	HR-MAS	3.77(t); 2.44(m); 2.12(m)
41	Guanosine	HR-MAS	7.99(s); 5.895(d);
42	Homocysteine	HR-MAS	3.87(dd); 2.68(m); 2.625(m);
43	Homocystine	HR-MAS	3.85(m); 2.82(m)
44	Methionine	HR-MAS	2.635(t); 2.125(s)
45	Nicotinamide	HR-MAS	8.93(d); 8.70(d); 8.24(m); 7.585(dd)
46	Uracil	HR-MAS	7.525(d); 5.795(d)
47	Uridine	HR-MAS	7.86(d); 5.91(d); 5.89(d);
48	Citrate	HR-MAS	2.69(d); 2.49(d)
49	N-acetylglutamine	HR-MAS	7.94(d); 4.15(m); 2.32(m); 2.02(s)
50	Tryptophan	HR-MAS	7.725(d); 7.54(d); 7.275 (t)
51	Arginine	Aq. Extracts	3.76(t); 1.91(m); 1.71(m); 1.62(m)
52	Aspartate	Aq. Extracts	2.80(dd); 2.675(dd)
53	GTP	Aq. Extracts	8.13(s); 5.93(d);
54	N-acetylcysteine	Aq. Extracts	2.935(dd); 2.90(dd); 2.07(s)
55	Succinate	Aq. extracts	2.39(s)
56	UMP	Aq. extracts	8.105(d); 5.99(d); 5.98(d)

Table S2: List of discriminant metabolites from volcano analysis (differentiated vs. PSC cells). Cutoff values considered are $|\log_2(FC)| > 1$ and $q < 0.05$. The Benjamini-Hochberg procedure to control the false discovery rate was applied for adjusting *p*-values (*q*-values).

HR-MAS Whole Cells

Metabolite	NMR shift (ppm)	FC	*q*-value
Uridine	5.885	2.561	0.00038
	7.865	3.2151	0.00126
	5.903	2.0272	0.00505
	5.871	5.9878	0.02983
Proline	3.322	2.2104	0.00673
Phenylalanine	7.430	2.2048	0.03590
Tryptophan	7.72	3.0908	0.03653
Unknowns	5.37	2.5402	0.00024
	1.104	2.3762	0.00145
	1.126	2.2353	0.00172
	5.082	2.5063	0.00180
	5.160	2.3258	0.02145
	4.590	3.8293	0.04082
Choline	3.198	-6.5256	4.1e-12
	3.504	-2.9255	2.4e-09
	3.525	-2.653	2.1e-09
GPC	3.684	-2.7797	2.3e-11
	3.604	-2.9107	6.4e-10
Creatine	3.923	-2.396	4.9e-09
UDP-X	5.966	-2.1229	4.4e-06
	5.539	-3.2508	0.02269
UDP-GlcNAc	5.507	-3.0294	3.1e-05
ADP/AMP	8.258	-2.3236	0.02565
AMP	8.561	-15.6353	5.3e-05
ADP	8.534	-10.0172	0.00251
Nicotinamide	7.585	-2.8217	0.03826
Unknowns	3.262	-2.522	2.3e-11
	2.755	-2.3861	8.3e-08
	8.060	-4.606	5.3e-05
	8.071	-2.1204	0.01626

Aqueous Extracts

Metabolite	NMR shift (ppm)	FC	*q*-values
Proline	2.026	2.2289	3.2e-08
	1.962	2.1466	3.2e-08
	1.998	2.5352	5.9e-08
	3.386	2.125	1.2e-06
	3.333	3.608	0.01389
Threonine	1.315	3.0145	3.2e-08
Isoleucine	1.211	2.2504	0.00016
	1.248	2.5084	0.00042
	1.447	2.157	5.9e-09
Inosine	4.428	14.4182	0.00048
Glutathione	4.543	12.1274	0.00828
	4.557	10.804	0.02614
Valine	2.242	6.3266	0.00013
	1.014	2.7283	3.2e-08
Unknowns	1.364	2.9232	3.2e-08
/ambiguous	3.464	2.1681	3.7e-06
	1.279	2.9954	0.00024
	0.858	2.0592	0.00372
	0.876	2.5368	0.00978
	2.757	2.0752	0.00157
	0.896	2.2817	0.01389
	0.751	2.264	0.04187
	4.462	2.6932	0.04296
Glycero-phosphocholine (GPC)	3.673	-4.7989	2.3e-07
	4.314	-6.5603	2.9e-07
	3.613	-2.9679	3.7e-07
	3.930	-3.4032	5.2e-07
	3.890	-2.9095	8.5e-07
	3.945	-2.5764	1.9e-06
	3.860	-2.0355	1.1e-05
Phosphocholine (PC)	3.208	-8.2205	2.3e-07
	3.584	-2.2826	2.9e-07
	4.156	-2.4667	2.5e-06
Creatine	3.914	-3.7177	5.2e-07
Choline	3.51	-2.3034	7.9e-06

	Taurine	3.261	-2.0223	1.0e-05
	Formate	8.445	-7.5978	0.00657
	Unknowns	3.014	-2.0381	5.5e-07
	/ambiguous	1.42	-2.0133	3.1e-05
